# Evolutionary and Functional Insights into Rice Universal Stress Proteins in Response to Abiotic Stresses

**DOI:** 10.3390/biology14101359

**Published:** 2025-10-03

**Authors:** Hong Lang, Yuxi Jiang, Yan Xie, Jiayin Wu, Yubo Wang, Mingliang Jiang

**Affiliations:** School of Agriculture, Jilin Agricultural Science and Technology College, Jilin 132101, China; lang_0916@163.com (H.L.); jiangyuxi0131@163.com (Y.J.); xieyancc05@163.com (Y.X.); 18333233134@163.com (J.W.); wyb070215@163.com (Y.W.)

**Keywords:** rice, universal stress protein, gene family evolution, expression profiling, abiotic stress adaptation

## Abstract

**Simple Summary:**

This study aimed to identify and characterize the Universal Stress Protein (USP) family in rice, a group of proteins known to mediate adaptation to harsh environments. We found 46 of these *OsUSP* genes in the rice genome and studied their characteristics, evolutionary relationships, and how they are turned on under stress. Our results show that these genes are activated by different stresses and are likely crucial for helping rice survive difficult conditions. Notably, *LOC_Os02g54590* and *LOC_Os05g37970* were strongly induced under all tested stress conditions, making them promising targets for genetic engineering and molecular breeding programs. This research provides a valuable foundation for future work aimed at improving the stress resistance of rice, offering potential strategies to develop more resilient rice varieties, which is vital for ensuring global food security in the face of climate change.

**Abstract:**

Universal Stress Protein (USP) plays crucial roles in plant stress adaptation, yet their evolutionary dynamics, regulatory mechanisms, and functional diversification in rice (*Oryza sativa*) remain poorly understood. This study aimed to conduct a genome-wide identification and characterization of the *OsUSP* gene family to elucidate its role in abiotic stress responses using integrated bioinformatics approaches. Here, we identified 46 *OsUSP* genes that are unevenly distributed across 11 rice chromosomes and exhibit significant divergence in protein length, molecular weight, and subcellular localization. Phylogenetic analysis classified *OsUSPs* into three subfamilies, with conserved motif and domain architectures within groups but distinct structural variations across subfamilies. Evolutionary analysis revealed strong collinearity between rice and other monocots, which suggests functional conservation in grasses, whereas limited synteny with dicots indicates lineage-specific divergence. *Cis*-regulatory element analysis showed enrichment in ABA, MeJA, drought, and hypoxia response motifs, implicating *OsUSPs* in hormonal and stress signaling. Expression profiling indicated tissue-specific patterns, with subfamily III genes broadly expressed, while subfamily II members were anther-enriched. Stress response profiling revealed that 24 *OsUSPs* were significantly induced, while *LOC_Os02g54590* and *LOC_Os05g37970* emerged as particularly notable due to their broad-spectrum responsiveness, being upregulated under all tested stress conditions. Protein–protein interaction (PPI) analysis indicated that OsUSP proteins potentially interact with Leo1/TPR-domain proteins and are involved in stress response and phosphorylation signaling pathways. This study yields key insights into OsUSP-mediated stress adaptation in rice and pinpoints promising candidate genes to facilitate the breeding of climate-resilient rice.

## 1. Introduction

Environmental stresses significantly impair both crop yield and quality. Among these, abiotic stress and soil nutrient deficiencies are the primary contributors to global agricultural losses, reducing average yields of major crops by over 50% [1]. Drought, salt, and extreme climatic conditions are the principal abiotic stressors, triggering a cascade of morphological, physiological, biochemical, and molecular disturbances that impair plant growth and development [2,3]. Rice (*Oryza sativa* L.), one of the most important food crops worldwide, is particularly sensitive to abiotic stress [4,5]. Reductions in rice yield caused by such stress are widespread and persistent. Moreover, the growing global population exacerbates challenges to food security [6]. Although significant progress has been made in identifying quantitative trait loci, genes, and signaling pathways involved in rice responses to abiotic stress, recent advances in functional genomics are accelerating the precision and scope of plant biotechnology. Therefore, identifying stress-related functional genes via whole-genome approaches is essential to elucidate the molecular mechanisms governing abiotic stress responses.

The universal stress protein (USP) family is a group of small molecular weight proteins that are widely distributed in bacteria, fungi, and plants, which primarily participate in plant growth, development, and cellular responses to biotic and abiotic stressors [7,8]. USPs were originally discovered in *Escherichia coli*, and studies have shown that *E. coli* USPs are classified into two subclasses based on structural similarity and amino acid sequence homology [9]. Class I, consisting of *UspA*, *UspC*, and *UspD*, lacks an ATP-binding motif, while Class II, which includes *UspF* and *UspG*, contains the ATP-binding motif [G-2X-G-9X-(S/T)] [9,10]. On the other hand, the number and structure of USPs in plants vary among different species. *USPs* are widely distributed in plants such as rice [11], *Arabidopsis thaliana* [12], *Solanum tuberosum* [13], *S. lycopersicum* [14], and other model plants. The structural complexity of plant USPs supports functional diversification through subtle motif variations. These catalytic motifs include serine/threonine kinases, tyrosine kinases, U-boxes, SWI2/snf2, homeodomain leucine zipper (HDzip), and C1 motifs insensitive to killer toxin 3 (IKI3) [7,15,16]. This functional diversity, which has been comprehensively reviewed across plant species by recent study [17], highlights the adaptability of *USPs* to diverse environmental stresses and their involvement in a wide range of physiological functions. Thus, a systematic and comprehensive analysis of the *OsUSP* family is necessary to elucidate their evolutionary history, regulatory mechanisms, and functional roles in abiotic stress adaptation in rice.

Recent studies have shown that several USP-like proteins show organ-specific expression patterns and play key regulatory roles in plant growth and development. In *A. thaliana*, the *AtUSP* gene (*At3g58450*) is implicated in seed germination and flowering; knockout mutants revealed delayed germination and inflorescence development [18,19]. Mutation of another *USP* gene, *HRU1*, results in phenotypes characterized by downward-curled leaves, reduced apical dominance, and altered root morphology, including modified lateral root formation [20]. *USPs* have also been linked to ethylene-mediated signaling, influencing fruit ripening [21]. In addition to developmental roles, *USP* genes are significantly upregulated under adverse environmental conditions. Plant *USPs* undergo rapid translational activation and cellular accumulation upon stress exposure, facilitating reprogramming of metabolic networks to promote tolerance and adaptation. Several reports have highlighted *USP* family members as key effectors in oxidative stress responses. *OsUSP1*, the first *USP* gene identified in rice, mediates ethylene-dependent adaptation to hypoxia [11]. In tomatoes, the *USP* gene *SlRd2* is phosphorylated by the protein kinase CIPK6 and participates in SlCipk6-mediated ROS generation [14]. Similarly, *SpUSP*, a *USP* gene cloned from wild tomatoes, is strongly induced by oxidative stress, and its overexpression mitigates oxidative damage [22]. Despite these significant findings, a systematic analysis of the entire *USP* family in rice is still lacking, which hinders a comprehensive understanding of their roles in stress adaptation and their potential application in breeding.

Beyond oxidative stress, *USP* gene expression is robustly induced by a spectrum of abiotic and biotic challenges. Under drought conditions, the transcription levels of *GUSP1* and *GUSP2* in *Gossypium* species increased over 200-fold in stressed leaves, and overexpression of *GUSP1* from *G. arboretum* was shown to enhance drought tolerance in transgenic cotton [23,24]. In response to salt, *USPs* are abundantly enriched in the roots of upland cotton [25]. Furthermore, *USPs* are implicated in responses to extreme temperatures and other abiotic stressors [26,27]. Their involvement also extends to biotic stress, including defense against pathogens [28] and parasitic nematodes [29]. Collectively, these studies clearly demonstrate that plant *USPs* are functionally responsive to a wide range of environmental threats; however, their precise biochemical and molecular mechanisms of action in rice remain largely uncharacterized.

Although Fan et al. (2024) [7] conducted a comparative study of *USP* gene family identification and expression profiles in *O. sativa*, *A. thaliana*, and *Zea mays*, a systematic analysis of the OsUSP family that integrates evolutionary dynamics, promoter architecture, protein interaction networks, and a comprehensive expression profiling under multiple abiotic stresses remains lacking. In this study, a systematic genome-wide identification and characterization of *USP* family members in rice was conducted via a combination of integrated bioinformatics approaches. Spatiotemporal expression patterns across major rice tissues and under different abiotic stress conditions were examined. Protein–protein interaction (PPI) network analysis was employed to investigate the signaling pathways associated with *USP* gene-mediated responses to abiotic stress. This research aims to bridge this knowledge gap and establishes a foundational framework for understanding the molecular basis of stress adaptation in rice and provides novel genetic targets for crop improvement programs.

## 2. Materials and Methods

### 2.1. Identification of OsUSP Genes in Rice

The protein sequences, CDS, genome assembly, and corresponding annotations of *O. sativa* ssp. *japonica* (cv. Nipponbare) was retrieved from the Ensembl Plants database (https://plants.ensembl.org) (accessed on 10 January 2025). A two-step approach was employed to identify *OsUSP* genes in the rice genome. First, the full-length protein sequences of *A. thaliana* USP genes were downloaded from TAIR and used as query sequences. Second, candidate sequences were screened using the Hidden Markov Model (HMM) profile for the USP domain (PF00582) obtained from the Pfam database (http://pfam.xfam.org/) (accessed on 13 January 2025). The HMM profile was reconstructed using HMMER3.4 and employed to search with an E-value  ≤  1 × 10^−5^ [13,15,30]. A BLASTp search (https://blast.ncbi.nlm.nih.gov/Blast.cgi) (accessed on 15 January 2025) was conducted to identify potential *USP* homologs in the rice genome using an E-value threshold of <1 × 10^−5^, consistent with the criteria used in previous family identification [31]. All alignments were manually checked to avoid the inclusion of questionable sequences. Candidate proteins containing the USP domain were validated via the NCBI Conserved Domain Database (CDD v3.20) following established criteria [13,15]. Corresponding gene sequences and CDS were extracted based on protein IDs.

Chromosomal positions were obtained based on physical location data and visualized using the Gene Structure View tool in TBtools (v2.152) [32]. Theoretical isoelectric point (pI), molecular weight (MW), and amino acid lengths of the OsUSP proteins were calculated using ExPASy ProtParam (https://web.expasy.org/protparam/) (accessed on 19 January 2025) [33]. Subcellular localization predictions were performed using a consensus approach integrating results from WoLF PSORT II (https://wolfpsort.hgc.jp/) (accessed on 22 January 2025), TargetP v2.0 (https://services.healthtech.dtu.dk/services/TargetP-2.0/) (accessed on 23 January 2025), and Plant-mPLoc v2.0 (http://www.csbio.sjtu.edu.cn/bioinf/plant-multi/) (accessed on 25 January 2025).

### 2.2. Multiple Comparisons and Phylogenetic Analysis

Full-length amino acid sequences of USP family proteins from rice and *A. thaliana* were aligned using MUSCLE (v3.8.425) implemented in MEGA11 [34]. Phylogenetic trees were constructed using the Neighbor-Joining (NJ) method with the Poisson correction model. Bootstrap analysis with 1000 replicates was performed to assess tree reliability. The evolutionary tree was graphically refined using the Interactive Tree of Life (iTOL; https://itol.embl.de/) (accessed on 29 January 2025).

### 2.3. Collinearity and Synteny Analysis of the OsUSP Genes

Collinearity and duplication events within the *OsUSP* gene family were assessed using Circos software v0.69-9 for visualization. Whole-genome duplication analysis was conducted via the Multiple Collinearity Scan Toolkit (MCScanX v1.0.0) with default parameters to identify segmental and tandem duplications. Genome sequence and gene annotation files for *O. rufipogon*, *Z. mays*, *S. viridis*, *S. bicolor*, *S. italica*, *A. thaliana*, *M. sativa*, *G. max*, *G. hirsutum*, and *S. tuberosum* were obtained from the Ensembl database. The Ka (non-synonymous substitution rate), Ks (synonymous substitution rate), and Ka/Ks ratios for duplicated gene pairs were calculated using Ka/Ks Calculator 2.0 [35] to infer selection pressure acting on duplicated *OsUSP* genes. The calculation incorporated the following parameters to ensure accuracy: gaps were treated as missing data, and Ka/Ks ratio > 1 and < 1 indicates positive selection and purifying selection, respectively.

### 2.4. Gene Structure, Conserved Domains, and Protein Motif Analysis

Gene structure information, including exon–intron boundaries and untranslated regions (UTRs), was extracted from the rice genome annotation file (GFF3 format). Protein motifs in rice USP protein sequences were predicted using MEME Suite (https://meme-suite.org/) (accessed on 5 February 2025) with the following settings: maximum number of motifs = 10; minimum motif width = 6; maximum motif width = 50. Conserved domains were verified using Pfam and NCBI-CDD annotations and visualized using TBtools. Gene structure, conserved motifs, and domains were integrated as key datasets for comparative analysis.

### 2.5. Cis-Acting Element Analysis

The 2000 bp upstream genomic sequences from the translation start site of each *OsUSP* gene were extracted and analyzed to identify putative *cis*-acting regulatory elements. The PlantCARE database (http://bioinformatics.psb.ugent.be/webtools/plantcare/html/) (accessed on 16 February 2025) was used to annotate and categorize the regulatory motifs located in the promoter regions.

### 2.6. Expression Patterns Analysis of OsUSP Genes

The developmental process of rice involves both vegetative and reproductive phases. Tissues representing vegetative growth include shoot apices, seedlings, roots, stems, leaves, and flag leaves, while reproductive growth involves tissues such as anthers, pistils, panicles, endosperms, milky seeds, and mature seeds. RNA-seq-based expression data (FPKM values) for these tissues and under various abiotic stress conditions were retrieved from the Sequence Read Archive (SRA) (https://www.ncbi.nlm.nih.gov/sra/) (accessed on 20 February 2025) and the Plant Public RNA-seq Database (https://plantrnadb.com/) (accessed on 22 February 2025). The data for tissue-specific expression obtained from the following publicly available datasets: DRX000523, DRX001075, ERX935621, SRX032239, SRX1219190, SRX1899050, SRX4881236, SRX4881241, SRX4881245, SRX507922, SRX507924, SRX507926, SRX507927. Data related to abiotic stress treatments (cold, heat, drought, and salt) were sourced from the datasets DRX120938, SRX5636330, SRX5636338, and SRX5636386. Expression data analysis was conducted in accordance with a previously established criteria [28]. Expression profiles were visualized using TBtools to generate heatmaps.

### 2.7. Plant Materials and Treatments

The rice variety ChangBai 9 (*O. sativa* L.) was used for abiotic stress experiments. This variety is considered a representative cultivar with moderate sensitivity to multiple abiotic stresses, making it a suitable model for investigating stress-responsive gene expression. Seeds were obtained from the experimental station of Jilin Agricultural Science and Technology College (Jilin, China). The preparation of the culture solution refers to the established method [36]. After germination, seedlings were cultivated in a controlled-climate growth chamber (RDN-1000D, Ningbo Yanghui, Ningbo, China) under a 16 h light/8 h dark photoperiod, with light provided by LED lamps (photon flux density: 250 μmol m^−2^ s^−1^; spectral range: 400–700 nm), at 28 °C and 65% relative humidity. Upon reaching the three-leaf stage, abiotic stress treatments were applied as follows: 150 mmol/L NaCl (S875253, Macklin, Shanghai, China) for salt stress; 4 °C for cold stress; 42 °C for heat stress; and 20% PEG 6000 (P815609, Macklin, Shanghai, China) for drought stress. All stress treatments and subsequent leaf sampling were carried out during the light period to maintain consistent physiological conditions. Leaf samples were collected at 0, 4, 8, 12, and 24 h after treatment and stored at −80 °C for subsequent analysis. Three biological replicates with at least five uniformly growing seedlings per replicate were sampled.

### 2.8. Quantitative PCR Validation

RNA extraction, reverse transcription, and quantitative PCR (qPCR) were conducted as previously detailed [36]. Gene-specific primers used for amplification are listed in Table S1. Relative expression levels were calculated using the 2^−ΔΔCt^ method [37]. The rice *Ubi* gene were used as internal reference to normalize gene expression data. All reactions were performed in triplicate.

### 2.9. PPI Network Prediction of OsUSP Proteins

The PPI network of rice USP proteins was predicted using the STRING database, with an interaction score threshold set to 0.7 (high confidence). The maximum number of interactors was limited to 10. The resulting PPI network was visualized using Cytoscape software v3.7.1.

### 2.10. Statistical Analysis

All statistical analyses were conducted using SPSS v22.0 (Chicago, IL, USA). One-way ANOVA followed by Duncan’s multiple range test was used to identify significant differences (*p* < 0.05). Data are presented as mean ± standard error. Pearson correlation analysis was conducted between chromosome length and the number of *OsUSP* genes per chromosome (*p* < 0.05).

## 3. Results

### 3.1. Identification and Molecular Characterization of USP Proteins in Rice

A total of 46 USP family members containing USP-like domains (PF00582) were identified in the rice (*O. sativa*) genome. These genes are unevenly and non-randomly distributed across 11 chromosomes (Table S2). No *OsUSP* genes were found on chromosome 4. The first five chromosomes (Chr. 1, Chr. 2, Chr. 3, Chr. 5, and Chr. 6) harbor 31 *OsUSP* genes, whereas chromosomes 6 through 12 each contain only 2–4 *OsUSP* genes. Statistical analysis using Pearson correlation coefficient revealed no significant correlation (*p* > 0.05) was observed between chromosome length and *OsUSP* gene distribution.

The encoded OsUSP proteins ranged from 164 (*LOC_Os03g22390*) to 1102 (*LOC_Os12g08060*) amino acids, with MW spanning 16,841.33 to 120,809.81 KDa and pI values ranging from 4.61 (*LOC_Os07g36600*) to 11.12 (*LOC_Os03g40130*), with an average of 7.07 (Table S2). The subcellular localization prediction analysis indicated that most OsUSP proteins were localized to the chloroplast, cytoplasm, and nucleus, except *LOC_Os01g07590* localized to both the mitochondria and chloroplast (Table S2). These results indicated that the significant sequence divergence among *OsUSP* genes may underlie their functional specialization in response to harsh environmental conditions.

### 3.2. Protein Conserved Motif, Domain, and Gene Structure Analysis of OsUSPs

**Figure 1:**
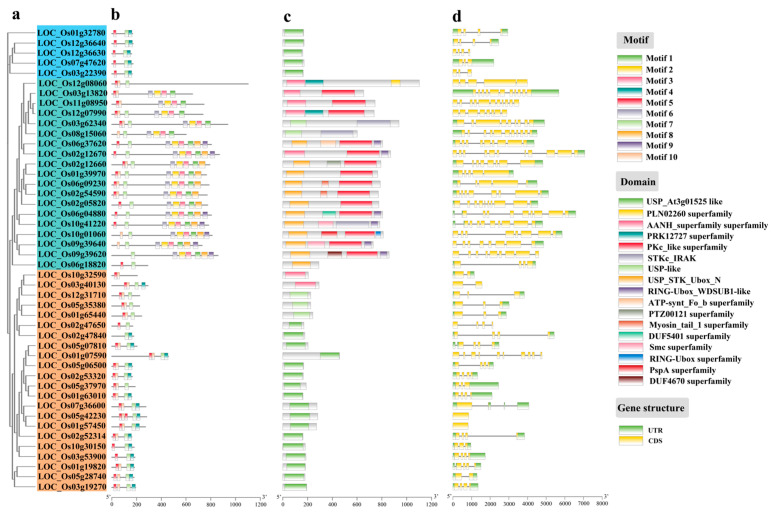
Phylogenetic and structural analysis of *OsUSP* genes and encoded proteins in rice. (**a**) The phylogenetic tree is constructed with the amino acid sequences of OsUSPs. Blue represents subfamily I. Green represents subfamily II. Yellow represents subfamily III. (**b**) Motif composition of rice OsUSPs. The motifs, numbers 1–10, are displayed in different colored boxes. (**c**) The domains of rice OsUSP proteins. Different colors represent different structural domains. (**d**) The exon–intron structure of *OsUSP* genes. Green boxes indicate untranslated 5′- and 3′- regions; yellow boxes indicate exons; black lines indicate introns. The X-axis in (**b**–**d**) represents the DNA size (bp).

Proteins, as the primary functional units in biological systems, provide essential insights into evolutionary divergence and functional specialization and functional diversification within gene families. To explore the structural diversity and evolutionary relationship among OsUSP proteins, a comprehensive analysis was performed, including phylogenetic classification, motif distribution, conserved domain identification, and exon–intron structural characterization. Based on the phylogenetic tree (Figure 1a), the 46 OsUSP proteins were classified into three subfamilies. Subfamily I comprised five members, representing the smallest group, whereas Subfamily II and Subfamily III included 19 and 22 members, respectively. Members of Subfamily II tended to encode longer peptides than those in Subfamilies I and III.

Motif distribution analysis revealed conserved patterns within subfamilies, indicating potential functional similarities (Figure 1b), while most proteins shared common motifs displayed subfamily specificity. For instance, Motifs 4, 5, and 7 were mainly distributed among Subfamily I, among which Motif 5 is located at the N-terminal of the most OsUSP protein sequence, and Motif 4 and Motif 7 are located at the C-terminal. There are conserved glycine residues associated with the USP domain in these three motifs. On the other hand, Subfamily II showed broader motif diversity with most members harboring 3–8 motifs, but shared core motifs with Subfamily I. Subfamily III exhibits similarity in motif pattern to Subfamily II, and typically contains Motifs 4, 5, 7, and 10. Conserved domain analysis (Figure 1c) indicated that most OsUSP proteins contain canonical USP-like or USP_AT3g01525-like domains. Subfamily I and III prominently exhibited these conserved domains, supporting potential functional conservation. Interestingly, some Subfamily II members (LOC_Os03g62340 and LOC_Os08g15060) contained USP-like domains, while several others possessed domains from the AANH_superfamily or USP_STK_Ubox_N, lacking the necessary motifs to constitute a complete USP_AT3g01525-like conserved domains. This diversity may suggest functional divergence within Subfamily II.

Gene structure analysis revealed variation in exon–intron organization among OsUSPs (Figure 1d). As expected, *OsUSPs* within the same group displayed similar numbers and arrangements of exons and introns and similar sequence features. The members of Subfamily I had 3–4 exons and 2–3 introns, with 2 genes (*LOC_Os12g36630* and *LOC_Os03g22390*) lacking 3′ UTR and 5′ UTR regions that may influence their post-transcriptional regulation or mRNA stability. However, significant differences in sequence length and the exon–intron number were observed in Subfamily II. The members of Subfamily II showed the widest structural variation, with exon numbers ranging from 4 to 11. In Subfamily III, most members possessed 1–4 exons and 1–3 introns except for LOC_Os01g07590, which had 10 exons and lacked 3′ and 5′ UTR regions. Among these members, the other four *OsUSPs* (*LOC_Os03g40130*, *LOC_Os02g47650*, *LOC_Os05g42230*, and *LOC_Os01g57450*) also lack 3′ and 5′ UTR regions. Most members contained introns, although *LOC_Os05g42230* and *LOC_Os01g57450* were intronless. In summary, OsUSP proteins display conserved motif, domain, and gene structures within subfamilies but notable divergence among them. The exceptional diversity of domain composition and gene structure in Subfamily III suggests its members may have evolved specialized roles in stress adaptation and regulatory processes.

### 3.3. Phylogenetic Analysis of OsUSPs in Rice

**Figure 2:**
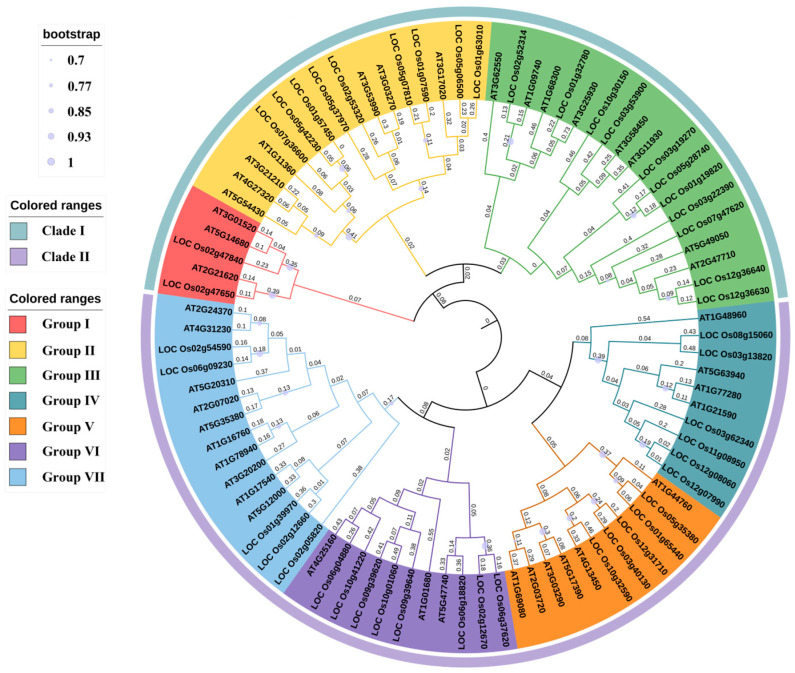
Phylogenetic analysis of USP proteins in rice and *A. thaliana*. The different-colored arcs indicate groups of the USP proteins. This tree was constructed using 46 OsUSP in rice and 41 AtUSP in *Arabidopsis*. The phylogenetic tree is built based on the complete amino acid sequences of the USP proteins by MEGA11 with the Neighbor-Joining method. Bootstrap = 1000. The solid circle on the main node represents the bootstrap values.

To investigate the evolutionary relationships of USP proteins in rice, a phylogenetic tree was constructed using full-length USP sequences from *O. sativa* and *A. thaliana* (Figure 2). A total of 87 USP proteins were classified into two major clades comprising seven groups (Groups I–VII). Clade I included Groups I–III, consisting of 22 *OsUSPs* and 18 *AtUSPs.* Members within each clade shared conserved motif composition and domain features, indicating a high degree of evolutionary conservation. The 22 OsUSPs in Clade I, all originating from Subfamilies I and III, were characterized by shorter peptides that typically featured motifs 4, 5, and 7, and possessed either USP_AT3g01525-like or USP-like domains. Clade II comprised the remaining groups, containing 24 *OsUSPs* and 23 *AtUSPs* (Figure 2). In contrast to Clade I, proteins in Clade II displayed more structural variability, particularly domain composition. Most *OsUSPs* in this Clade encoded longer peptides and contained an additional kinase domain along with the USP domain. Analysis of the complete phylogenetic tree indicated that USP proteins from rice and *A. thaliana* were interspersed across the clades, without clear species-specific clustering, implying conserved evolutionary patterns across species.

### 3.4. Collinearity Analysis of OsUSPs in Rice

**Figure 3:**
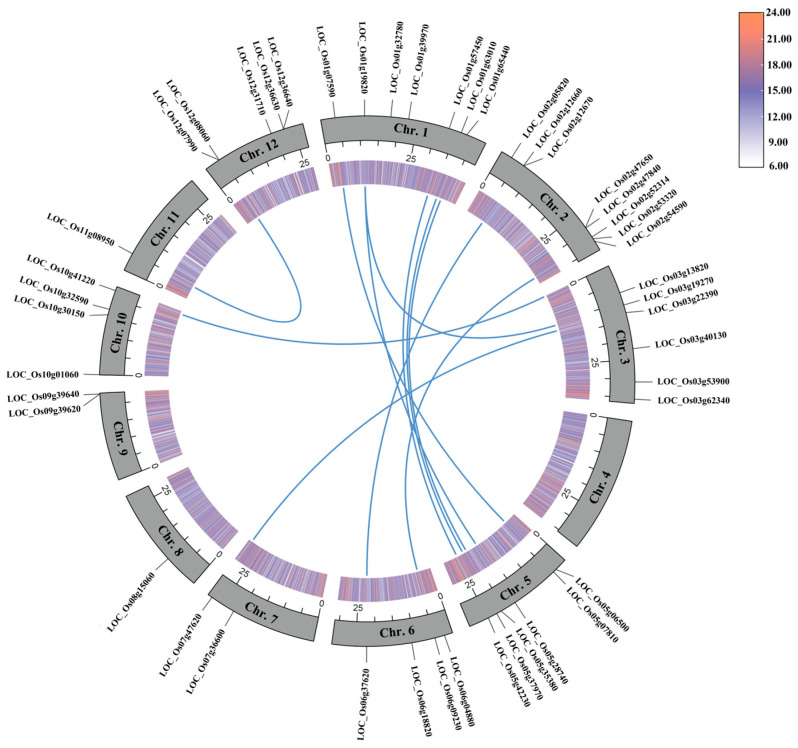
The synteny analysis of *OsUSPs* in the rice genome. The rings from the inside out represent the gene density heat map (orange for high gene density; purple for low gene density) and chromosome skeleton. The collinear pairs of *OsUSPs* genes in the rice genome are highlighted by colored lines.

Segmental duplication and tandem duplication are two major duplication mechanisms driving gene family expansion. Collinearity analysis was performed on all rice *USP* genes to assess the duplication events of *OsUSP* (Figure 3). A total of 21 *OsUSPs* were found to be collinear region, with 11 collinear pairs segmental duplication. Chr. 1 and Chr. 5 show the maximum number of collinear genes, with five gene pairs being collinear. However, Chr. 2, Chr. 3, and Chr. 6 had 2, 3, and 2 collinear genes, respectively, while Chr. 7, Chr. 10, Chr. 11, and Chr. 12 each contained only one collinear *OsUSP* gene (Figure 3). Multiple types of genomic distribution were detected in rice’s *USP* genes, and the dispersed duplication event (50.00%) was the most common pattern (50.00%), while tandem and proximal duplications also contributed substantially, accounting for 28.26% and 21.74% of the distribution, respectively.

Selection pressure on *OsUSP* paralogs was assessed by calculating Ka, Ks, and Ka/Ks ratios. The Ka/Ks values ranged from 0.1349 to 1.0313 (Table S3), and most *OsUSP* genes were subjected to strong purifying selection (Ka/Ks < 1) following gene duplication, indicating potential functional redundancy under specific conditions. One gene pair (*LOC_Os03g22390*/*LOC_Os07g47620*) showed evidence of positive selection (Ka/Ks > 1), suggesting divergence at the protein level during stress-associated evolutionary processes. These results indicate a combination of selective pressures, with predominant functional conservation and occasional adaptive divergence among OsUSP proteins.

To elucidate the evolutionary mechanisms and duplication events of *OsUSP* genes, comparative synteny analysis was performed between *O. sativa* and ten representative plant species, including five monocotyledons (*O. rufipogon* Griff, *Z. mays* L., *Setaria viridis* L., *Sorghum bicolor* L., and *Setaria italica*) and five dicotyledons (*A. thaliana*, *Glycine max* L., *Medicago sativa* L., *Gossypium hirsutum* L., and *S. tuberosum* L.).

**Figure 4:**
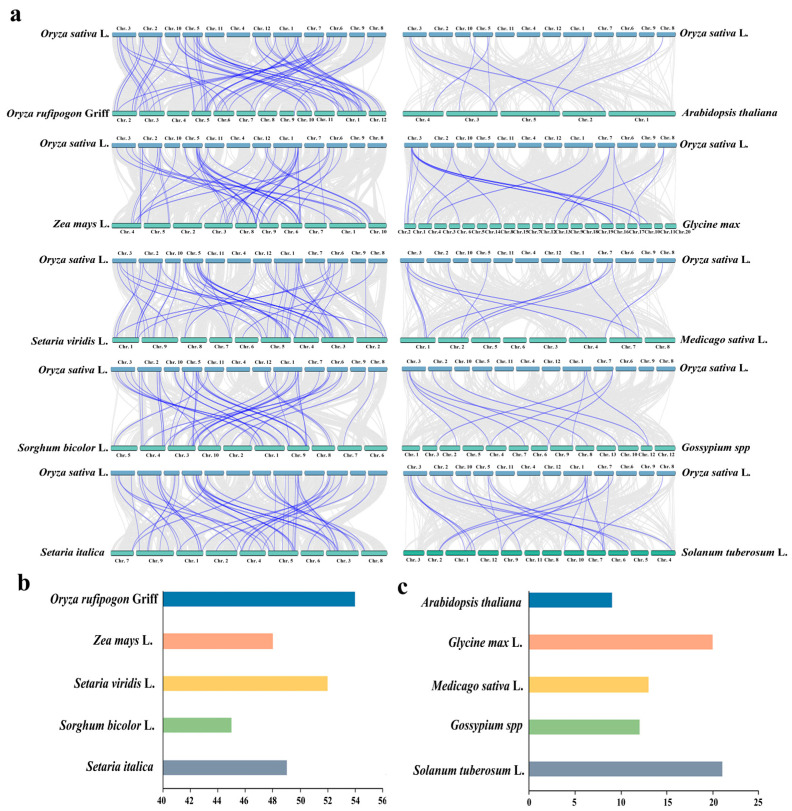
Synteny analysis of *USP* genes between rice and other plants. (**a**) Collinearity analysis of *USP* genes between *O. sativa* and *O. rufipogon* Griff, *Z. mays* L., *S. viridis* L., *S. bicolor* L., *S. italica*, *A. thaliana*, *G. max* L., *M. sativa* L., *G. hirsutum* L., and *S. tuberosum* L. The gray background indicates all the collinear blocks, the gray background lines represent all the collinearity modules, and the blue lines represent gene pairs with collinearity. (**b**) The number of pairs of *USP* collinearity genes between rice and grass crops. (**c**) The number of pairs of *USP* collinearity genes between rice and other plants. The X-axis in (**b**,**c**) represents the number of gene pairs.

The *USP* genes on different chromosomes of rice are relatively conserved in monocots. There are 54, 48, 52, 45, and 49 pairs of collinear genes in wild rice, maize, *S. viridis*, sorghum, and foxtail millet, respectively (Figure 4a,b). These collinear genes of *USP* retain conserved genomic positions across major grass crops because they are all diploid organisms. It is worth mentioning that rice has more collinear genes of *USP* with wild rice than other crops, indicating that the *USP* genes have not been eliminated in the rice domestication and selection process, further suggesting that the *USP* gene has important biological functions in rice stress response.

To further explore the evolutionary conservation of *USP* genes across diverse plant lineages, a comprehensive collinearity relationship was performed between rice and five representative species spanning distinct taxonomic families, including *A. thaliana* (Brassicaceae), alfalfa and soybean (Fabaceae), cotton (Malvaceae), and potato (Solanaceae). In the collinearity analysis with the *Arabidopsis* genome, only 9 direct orthologs of *OsUSP* were identified (Figure 4c). On the other hand, in the alfalfa, soybean, cotton, and potato genomes, 20, 13, 12, and 21 homologs were identified, respectively, suggesting that rice and *Arabidopsis* show significant evolutionary divergence. Despite such deep phylogenetic divergence, significant conserved features were observed between monocotyledons and dicotyledons. These analyses suggest that duplicated *USP* gene pairs exhibit significant evolutionary and functional relationships.

### 3.5. Characterization of Cis-Acting Elements in OsUSP Promoters Regions

**Figure 5:**
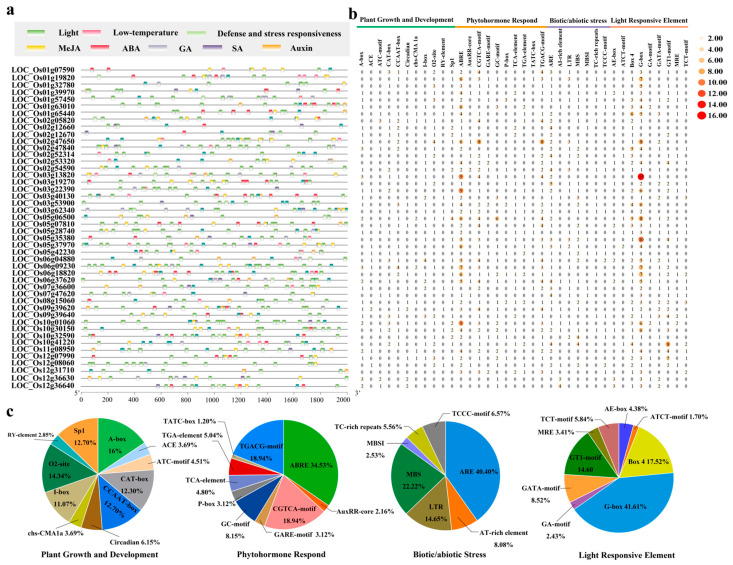
*Cis*-element analysis of *OsUSP* gene promoters. (**a**) *Cis*-acting elements in the 2000 bp upstream region of the *OsUSP* gene promoters. (**b**) Distribution of *cis*-acting elements of *OsUSP* gene members. The color scale represents high (red) to low (orange) quantity levels. (**c**) The ratio of *cis*-acting elements associated with the four biological categories. Different colors depict the various *cis*-elements.

To explore the regulatory mechanisms underlying *OsUSP* gene expression, 2 kb upstream promoter sequences were retrieved for all identified *OsUSP* genes, and *cis*-acting regulatory elements were predicted using the PlantCARE database (Figure 5a; Table S4). Various *cis*-elements associated with light responsiveness, phytohormonal regulation (MeJA, ABA, GA, SA, and auxin), and abiotic/biotic stress responses were identified. These elements were categorized into four functional groups: (I) plant growth and development, (II) phytohormone response, (III) biotic/abiotic stress response, and (IV) light responsiveness (Figure 5b). Hormone-responsive elements were particularly enriched, with ABA-responsive ABRE motifs accounting for 34.53% and MeJA-responsive CGTCA- and TGACG-motifs comprising 37.88% of hormone-related elements (Figure 5c; Table S4). Other elements responsive to GA, SA, and auxin were also detected, suggesting broad hormonal regulation of *OsUSP* genes. Stress-related *cis*-elements were prominent in *OsUSP* promoters, including hypoxia-responsive ARE (40.40%), drought-inducible MBS elements (22.22%), low-temperature responsive LTR elements (14.65%), and TC-rich repeats associated with defense and general stress responses (5.56%) (Figure 5b,c; Table S4). The significant enrichment of stress- and hormone-responsive motifs provides strong evidence for the role of *OsUSP* genes in environmental stress adaptation. Furthermore, the presence of growth- and light-related elements indicates the potential involvement of *OsUSPs* in diverse developmental and physiological processes.

### 3.6. Expression Patterns of OsUSPs in Different Tissues and Under Abiotic Stress Conditions

**Figure 6:**
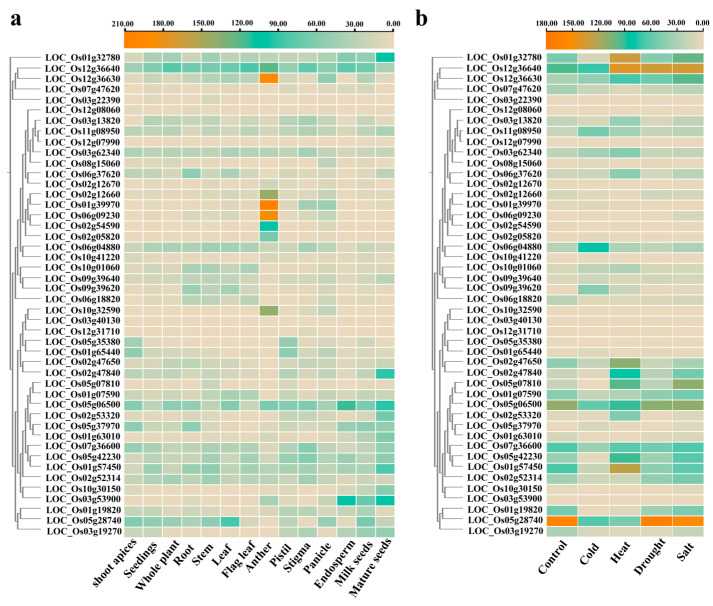
Expression patterns of *OsUSP* genes in different tissues and under stress conditions. (**a**) The expression patterns of *OsUSP* genes in 12 tissues. (**b**) Expression patterns of *OsUSP* genes under cold, heat, drought, and salt. The expression values were mapped using a color gradient from pink to orange, as shown at the top of the heatmap.

To elucidate the functional roles of *OsUSP* genes, RNA-seq data were analyzed to quantity their expression profiles across major rice tissues and under various abiotic stress conditions based on FPKM values (Table S5). As shown in Figure 6a, *OsUSP* genes exhibited distinct spatial expression patterns with subfamily-specific differences. In Subfamily I, *LOC_Os01g32780* and *LOC_Os12g36640* were highly expressed across all tissues, while *LOC_Os12g36630* showed elevated expression specifically in anthers. Subfamily II members displayed organ-specific expression; for example, *LOC_Os02g12660*, *LOC_Os01g39970*, *LOC_Os06g09230*, *LOC_Os02g54590*, and *LOC_Os02g05820* were mainly expressed in anthers, suggesting potential involvement in reproductive development. Subfamily III members were broadly expressed across tissues, with 15 out of 22 genes exhibiting high transcript abundance. On the other hand, two genes, *LOC_Os03g40130* and *LOC_Os12g31710*, showed negligible expression in all tissues.

To access stress-responsive dynamics, expression profiles under cold, heat, drought, and salt stress were examined (Figure 6b; Table S6). A total of 17, 24, 20, and 24 *OsUSP* genes were upregulated under cold, heat, drought, and salt stress, respectively. Distinct patterns of transcriptional regulation were observed across stress treatments. For instance, *LOC_Os12g36640* showed increase under heat, drought, and salt (with FPKM values increasing from 94.67 to 140.03, 133.77, and 135.37, respectively) but was decreased under cold stress (FPKM decreased from 94.67 to 71.40). Five genes (*LOC_Os12g36630*, *LOC_Os11g08950*, *LOC_Os10g01060*, *LOC_Os09g39640*, and *LOC_Os09g39620*) were consistently upregulated across multiple stress conditions, indicating potential roles as core regulators in abiotic stress responses. On the other hand, stress significantly inhibited the expression of several *OsUSP* genes, including *LOC_Os07g47620*, *LOC_Os06g18820*, *LOC_Os05g35380*, *LOC_Os01g63010*, *LOC_Os01g19820*, and *LOC_Os03g19270*, their consistent downregulation suggests they may act as negative regulators of stress responses, possibly to prevent overactivation of defense mechanisms or to maintain cellular homeostasis under adverse conditions.

**Figure 7:**
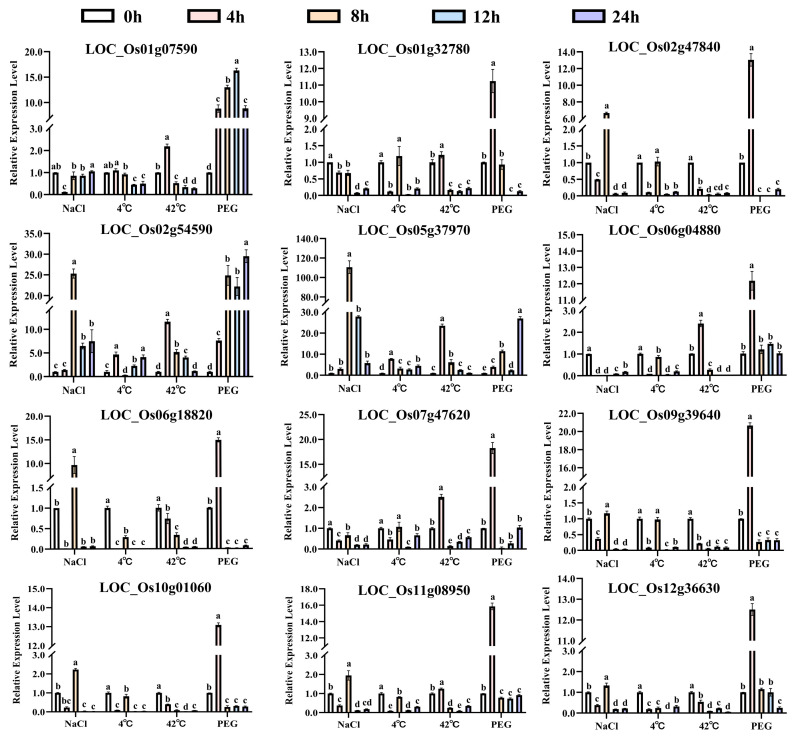
Spatiotemporal expression patterns of *OsUSP* genes in leaf after NaCl, 4 °C, 42 °C, and PEG treatments. Statistically significant differences (*p* < 0.05, one-way ANOVA) are denoted by different lowercase letters. Data are presented as mean ± SE from three biological replicates.

Twelve genes from three subfamilies were selected for further validation of their expression patterns based on transcriptomic data showing the most significant differential expression under abiotic stress conditions (Figure 7). The expression levels of twelve *OsUSPs* reveal a good correlation with the mRNA-seq data and show differential expression patterns in response to various abiotic stresses. For instance, *LOC_Os01g07590* showed initial suppression from 4 to 12 h, followed by a significant increase (*p* < 0.05) at 24 h under drought stress. Most *OsUSP* genes displayed dynamic and stress-specific expression kinetics. Under salt treatment, ten genes reached maximal expression at 8 h, with *LOC_Os05g37970* showing a nearly 110-fold upregulation (Figure 7). Seven genes responded strongly to cold stress; another seven were significantly induced and peaked at 4 h post-heat exposure. Under drought, all twelve selected genes demonstrated higher expression than under the other stress conditions. *LOC_Os02g54590* and *LOC_Os05g37970* were significantly induced under all four stress treatments compared to those in the control group, while *LOC_Os05g35380* was consistently suppressed under the same conditions. These results demonstrate that *OsUSP* genes exhibit coordinated and dynamic regulation under abiotic stress, functioning as key molecular hubs in rice stress adaptation.

### 3.7. Protein–Protein Interaction (PPI) Network of OsUSP in Rice

**Figure 8:**
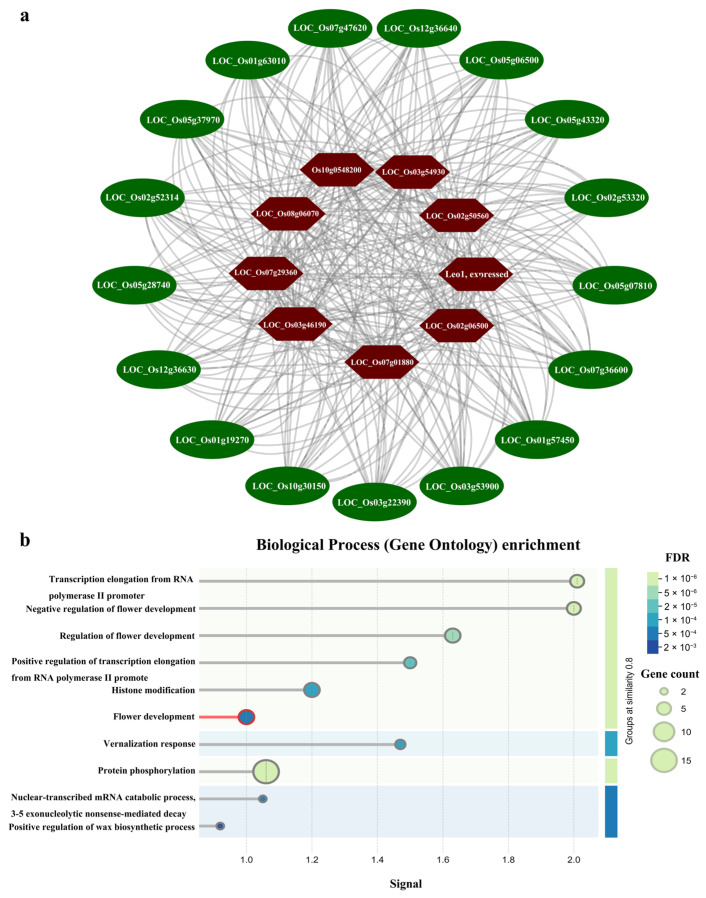
Protein–protein interaction network of OsUSP and GO enrichment analysis. (**a**) Protein interaction network of rice OsUSPs. Green and rednodes represent rice OsUSPs and interaction proteins, respectively. (**b**) GO enrichment analysis of proteins in the PPI network.

A PPI network was constructed to further investigate the potential regulatory mechanisms of OsUSP in rice (Figure 8). Central nodes in the network included proteins containing Leo1 and tetratricopeptide repeat (TPR) domains, including LOC_Os03g54930, LOC_Os07g01880, and Os10g0548200, which may participate in stress responses through interactions with OsUSP proteins (Figure 8a). For example, the Os10g0548200 (BSR-K1) gene encodes a TPR-domain RNA-binding protein; its functional loss has been associated with increased resistance to two key rice pathogens [38]. GO enrichment analysis of the PPI network indicated that OsUSP proteins are functionally associated with key pathways involved in flower development, histone modification, vernalization response, protein phosphorylation, wax biosynthetic process, and other cellular processes (Figure 8b). These findings align with the known roles of universal stress proteins in regulating cross-talk between multiple signaling pathways during plant adaptation to stress. These results highlight the multifaceted regulatory functions of *OsUSP* genes and suggest their involvement in broad cellular networks modulating plant growth, development, and abiotic stress responses. It should be noted that the PPI network presented here is based on in silico predictions with high confidence, and future experimental validation such as yeast two-hybrid (Y2H) or co-immunoprecipitation (Co-IP) assays will be necessary to confirm these interactions.

## 4. Discussion

### 4.1. Distribution of USPs in Plants

Rice is a staple crop that feeds approximately half of the world’s population, making it critical to global food security [39,40]. Abiotic stresses, including drought, salt, and temperature extremes, are major environmental factors that severely limit rice yield and quality [2]. *USP* genes form an evolutionarily conserved protein present across the prokaryotes and eukaryotes domains, with significant expansion in higher plants. These genes play essential roles in growth, regulation, development, and adaptive response to abiotic stress conditions. Therefore, a comprehensive characterization of *USP* genes in rice is a key step toward enhancing stress resilience and ensuring yield stability in rice production systems.

Previous studies have revealed that *USP* gene family size varies considerably across different plant species. There are 41, 44, 23, 25, 88, 51, and 108 *USPs* identified in *Arabidopsis*, rice [41], maize [7], barley [42], wheat [15], pigeonpea [43], and potato [13], respectively. Furthermore, comparative genomics revealed strikingly different numbers of USP genes among *Gossypium* species. For instance, a total of 49, 52, 102, and 104 *USP* genes were identified in *G*. *arboretum* (A2, 2n = 2x = 26), *G*. *raimondii* (D5, 2n = 2x = 26), *G*. *hirsutum* (AD1, 2n = 4x = 52), and *G*. *barbadense* (AD2, 2n = 4x = 52) [44]. The number of USP genes reported in rice has also varied across studies. Previous studies by Arabia et al. (2021) [41] and Fan et al. (2024) [7] identified 44 and 43 *USP* family members, respectively. These interspecies and intraspecies variations likely reflect differences in genome annotation quality, gene prediction algorithms, and filtering criteria used across studies. In this study, *46 OsUSP* genes were identified in the rice Nipponbare genome, including two previously unreported members, *LOC_Os03g40130* and *LOC_Os06g18820*. The stringent identification process employed in this study, which combined a two-step BLASTp and HMMER search with manual NCBI-CDD validation, may explain the differences in gene count compared to previous works. No consistent correlation was observed between genome size and *USP* gene family size across species. The 46 *OsUSP* genes were found to be unevenly distributed across 11 of 12 rice chromosomes, each exhibiting distinct physicochemical characteristics (Table S2). Phylogenetic tree reconstruction revealed that the OsUSP proteins could be grouped into two major clades and eight subgroups, consistent with previous studies (Figure 2). As previously reported, members of Clade II typically exhibited longer peptide lengths, suggesting possible functional divergence [41].

Gene duplication analysis indicated that segmental and tandem duplications were the primary mechanisms underlying *OsUSP* gene family expansion. Synteny analysis further revealed strong evolutionary conservation between cultivated rice (Nipponbare) and wild rice (W1943), with 54 syntenic *USP* gene pairs identified (Figure 4). The conservation of *USP* genes during rice domestication argues for their fundamental biological importance. This strong synteny further suggests that wild rice germplasm represents a valuable reservoir of allelic variation, harnessing which could enhance stress resilience in modern breeding programs. Comparative collinearity analysis across monocots and dicots revealed higher orthology levels between *OsUSP* genes and those in wild rice, *S. viridis*, and *S. italica*, compared to *A. thaliana*, reflecting both evolutionary conservation and lineage-specific divergence patterns (Figure 4). These findings support the notion that the *USP* gene family underwent lineage-specific differentiation following the monocot–dicot split, a process likely driven by functional specialization and adaptation to distinct environmental pressures in cereals. The expansion and retention of specific *OsUSP* subfamilies in rice could be crucial for its adaptation to abiotic stresses common in agroecosystems.

### 4.2. Structural and Functional Diversity of USPs

#### 4.2.1. Phylogenetic Classification and Domain Architecture Underpin Functional Diversification

The significant structural variation observed among the 46 identified *OsUSP* genes underscores both evolutionary adaptation and potential functional diversification. Phylogenetic classification revealed three distinct subfamilies (I–III), each displaying conserved gene structures and motif arrangements within the subgroups despite considerable diversity at the family level (Figure 1). Members of subfamilies I and III have single USP-like or USP-At3g01525-like domains, respectively. On the other hand, subfamily II proteins possess one or two tandem USP domains in combination with additional functional domains such as AANH_superfamily, PKc_like superfamily, USP_STK_Ubox_N, and STKc_IRAK. This domain architecture supports a wider functional range and parallels the domain composition of USP proteins in wheat [15] and potato [13]. The presence of additional functional domains in subfamily II OsUSPs implies their potential involvement in more complex regulatory pathways in response to abiotic stress.

#### 4.2.2. *Cis*-Regulatory Elements Reveal Hormonal and Stress-Responsive Regulation

The *cis*-regulatory elements located in gene promoter regions serve as critical determinants of spatiotemporal gene expression patterns [45]. Promoter analysis of *OsUSP* genes revealed a significant enrichment of *cis*-acting elements associated with abiotic stress responses, such as ARE (involved in anaerobic induction), MBS (linked to drought responsiveness), and LTR (related to low-temperature responsiveness) (Figure 5). These findings are consistent with previous studies demonstrating *USP* involvement in heat, salt, dehydration, osmotic, and cold stress responses. For instance, *Arabidopsis AtUSP* (*At3g53990*) has been demonstrated to act as a molecular chaperone during heat and oxidative stress [46], as well as an RNA chaperone that stabilizes secondary structures under cold stress [47]. The same gene exhibited transcriptional induction under salt, osmotic, and mechanical stress [26]. Furthermore, *AtPHOS32* (*At5g54430*) and *AtPHOS34* (*At4g27320*) have been shown to undergo phosphorylation in response to microbial elicitation [48]. Functional links to abiotic stress tolerance have also been established for *USP* family members in key crop species, including cotton, tomato, and rice [11,23]. These findings may position *OsUSP* genes as key regulators in the transcriptional network controlling plant stress adaptation.

#### 4.2.3. Integrated Structural and Regulatory Features Support Roles in Stress Adaptation

Phytohormones such as ABA and MeJA play essential roles in mediating plant responses to environmental stress [49,50]. Multiple studies have reported hormone-induced regulation of *USP* gene expression. For example, *At3g58450* expression increased more than 35-fold in response to 100 µM ABA but was unaffected by 10 µM GA treatment [18]. Consistently, *AtUSP17*-overexpressing lines exhibited enhanced ABA sensitivity compared to knockdown mutants [51]. In cotton, promoter activity of *GUSP* was significantly induced by both ABA and GA [52]. Similarly, in rice, *OsUSP1* has been shown to participate in ethylene-mediated hypoxia signaling [11]. Consistent with these findings, our cis-regulatory analysis revealed a pronounced enrichment of ABA-responsive (ABRE) and MeJA-responsive (CGTCA/TGACG-motif) elements in *OsUSP* promoters, strongly implicating their functional integration into well-defined stress-signaling cascades. Specifically, ABRE motifs suggest involvement in ABA-dependent signaling pathways mediated by SnRK2 kinases and other transcription factors [36,52]. These connections posit *OsUSPs* as potential downstream effectors or modulators within these hormonal networks, translating canonical stress signals into adaptive physiological responses. Taken together, multi-dimensional evidence from gene structure, motif architecture, regulatory elements and hormone responsiveness highlights the structural and functional complexity of the rice *USP* family, demonstrating their role as critical hubs in abiotic stress adaptation networks through convergent regulatory mechanisms.

### 4.3. Potential Molecular Mechanism Underlying Abiotic Stress Responses of USPs

Comparative analysis of gene expression across various tissues and abiotic stress conditions revealed significant expression plasticity among *OsUSP* family members, suggesting functional differentiation in response to environmental stimuli. Five *OsUSP* genes exhibited consistent upregulation, while three were consistently downregulated under all stress treatments examined. qPCR validation confirmed the dynamic expression of selected *OsUSP* genes under drought, salt, cold, and heat stress, consistent with the conserved stress-responsive nature of *USP* families reported in other plant species. For instance, *USP* expression in wheat is tightly regulated in a tissue-specific manner under heat stress [15], while transcriptomic analyses in potatoes indicated *USP* involvement in mechanical damage and deoxynivalenol stress responses [13]. These observations reinforce the broad functional relevance of *USP* genes in plant adaptation to diverse environmental pressures.

A PPI network was constructed to further elucidate the molecular mechanisms underlying *OsUSP*-mediated stress responses. Several key interactors, such as LOC_Os03g54930, LOC_Os07g01880, and Os10g0548200, were identified as potential regulatory nodes associated with stress-related signaling pathways. These candidate genes warrant further investigation to clarify their roles in *OsUSP*-mediated stress adaptation.

### 4.4. Future Research Directions

Although this study provides a comprehensive genomic and transcriptomic framework for *OsUSP* gene characterization, the predicted mechanisms of action require experimental confirmation. Our PPI network analysis suggests OsUSPs may interface with phosphorylation cascades and transcriptional regulation, providing concrete hypotheses for their mechanistic roles in stress signaling. Furthermore, the significant enrichment of ABA and MeJA responsive elements in the promoters strongly suggests the functional integration of these *OsUSPs* into core hormonal signaling pathways that mediate abiotic stress responses. The subcellular localizations of the identified OsUSP proteins, predicted in this study, require experimental confirmation through techniques such as confocal microscopy of fluorescently tagged proteins to unequivocally determine their in planta compartmentalization.

Future research should employ techniques such as CRISPR/Cas9-mediated gene editing, transgenic overexpression, and RNA interference (RNAi) to elucidate the specific functions of individual *OsUSP* genes, validate the predicted protein interactions, and verify their contributions to abiotic stress tolerance through these proposed mechanisms. Functional characterization of these candidates (such as *LOC_Os02g54590* and *LOC_Os05g37970*) will be critical for assessing their potential as genetic resources for breeding stress-resilient rice cultivars. While this study provides foundational insights through in silico analyses in a single cultivar, future work incorporating diverse genetic backgrounds and experimental validation will be essential to generalize the findings and elucidate the precise functions of *OsUSPs* in stress adaptation.

## 5. Conclusions

This study presents a comprehensive genomic and functional characterization of the *USP* family in rice, identifying 46 *OsUSP* genes. Phylogenetic analysis classified them into three subfamilies exhibiting distinct conserved motifs and gene structures. *Cis*-element analysis revealed a significant enrichment of stress- and hormone-responsive motifs in their promoters. Expression profiling demonstrated that 24 *OsUSP* genes were significantly induced under various abiotic stresses, with *LOC_Os02g54590* and *LOC_Os05g37970* emerging as core broad-spectrum responsive regulators, being upregulated under all tested conditions. OsUSPs are predicted to interact with Leo1/TPR-domain proteins and participate in stress response and phosphorylation pathways. Collectively, these findings enhance our understanding of *OsUSP*-mediated stress adaptation mechanisms and establish a genetic foundation for future efforts to improve abiotic stress tolerance in rice through molecular breeding strategies.

## Data Availability

All relevant data are available within the article and its Supplementary Data.

## Supplementary Materials

The following supporting information can be downloaded at: https://www.mdpi.com/article/10.3390/biology14101359/s1, Table S1: List of primer pairs used for qPCR analysis; Table S2: Information of *OsUSP* family genes identified in rice; Table S3: Calculating the Ka, Ks, and Ka/Ks values; Table S4: *Cis*-acting elements in the 2000 bp upstream region of *OsUSP* gene promoters; Table S5: Tissue-specific expression patterns of *OsUSP* genes; Table S6: Stress-responsive profiles of *OsUSP* genes.

## Abbreviations

The following abbreviations are used in this manuscript:

CDD	Conserved Domain Database
Co-IP	Co-immunoprecipitation
Ka	Non-synonymous substitution rate
Ks	Synonymous substitution rate
MW	Molecular weight
pI	Theoretical isoelectric point
PPI	Protein–protein interaction
qPCR	Quantitative PCR
SRA	Sequence Read Archive
TPR	Tetratricopeptide repeat
USP	Universal Stress Protein
UTRs	Untranslated regions
Y2H	Yeast two-hybrid
